# Molecular Mechanisms and Potential Clinical Applications of *Campylobacter jejuni* Cytolethal Distending Toxin

**DOI:** 10.3389/fcimb.2016.00009

**Published:** 2016-02-09

**Authors:** Cheng-Kuo Lai, Yu-An Chen, Chun-Jung Lin, Hwai-Jeng Lin, Min-Chuan Kao, Mei-Zi Huang, Yu-Hsin Lin, Chuan Chiang-Ni, Chih-Jung Chen, U-Ging Lo, Li-Chiung Lin, Ho Lin, Jer-Tsong Hsieh, Chih-Ho Lai

**Affiliations:** ^1^Department of Microbiology and Immunology, Graduate Institute of Biomedical Sciences, College of Medicine, Chang Gung UniversityTaoyuan, Taiwan; ^2^Department of Urology, University of Texas Southwestern Medical CenterDallas, TX, USA; ^3^School of Medicine, Graduate Institute of Basic Medical Science, China Medical UniversityTaichung, Taiwan; ^4^Department of Internal Medicine, School of Medicine, College of Medicine, Taipei Medical UniversityNew Taipei, Taiwan; ^5^Division of Gastroenterology and Hepatology, Department of Internal Medicine, Shuang-Ho HospitalNew Taipei, Taiwan; ^6^Department of Biological Science and Technology, China Medical UniversityTaichung, Taiwan; ^7^Department of Pediatrics, Molecular Infectious Disease Research Center, Chang Gung Children's Hospital and Chang Gung Memorial HospitalTaoyuan, Taiwan; ^8^Department of Life Sciences, National Chung Hsing UniversityTaichung, Taiwan; ^9^Graduate Institute of Cancer Biology, China Medical UniversityTaichung, Taiwan; ^10^Department of Nursing, Asia UniversityTaichung, Taiwan

**Keywords:** *Campylobacter jejuni*, cholesterol, cytolethal distending toxin, enzymatic activity, trafficking

## Abstract

Cytolethal distending toxin (CDT), a genotoxin produced by *Campylobacter jejuni*, is composed of three subunits: CdtA, CdtB, and CdtC. CdtB is a DNase that causes DNA double-strand breaks (DSB) in the nucleus resulting in cell cycle arrest at the G2/M stage and apoptosis. CdtA and CdtC bind to cholesterol-rich microdomains on the cytoplasmic membrane, a process required for the delivery of CdtB to cells. Although a unique motif associated with cholesterol-binding activity has been identified in other pathogens, the mechanism underlying the interaction between the CdtA and CdtC subunits and membrane cholesterol remains unclear. Also, the processes of cell uptake and delivery of CdtB in host cells and the translocation of CdtB into the nucleus are only partially understood. In this review, we focus on the underlying relationship among CDT, membrane cholesterol, and the intracellular trafficking pathway as a unique mechanism for *C. jejuni*-induced pathogenesis. Moreover, we discuss the clinical aspects of a possible therapeutic application of CDT in cancer therapy. Understanding the molecular mechanism of CDT-host interactions may provide insights into novel strategies to control *C. jejuni* infection and the development of potential clinical applications of CDT.

## Introduction

*Campylobacter jejuni*, a gram-negative bacterium, is one of the most common causative agents of food-borne infectious illnesses in humans (Butzler and Skirrow, 1979; Mead et al., 1999). Infection by *C. jejuni* in humans usually occurs through the consumption of contaminated poultry products (Corry and Atabay, 2001), and *C. jejuni*-associated enterocolitis is typically associated with a local acute inflammatory response that involves intestinal tissue damage (Black et al., 1988). An important *C. jejuni* virulence factor, cytolethal distending toxin (CDT), has been characterized in detail (Lara-Tejero and Galan, 2000) and is thought to be associated with *C. jejuni*-induced local acute inflammation involved in enterocolitis (Zheng et al., 2008). Although CDT can be produced by various gram-negative bacteria with certain diversity in genes, its ultimate function resembles that of a genotoxin (Gargi et al., 2012). The interactions between *C. jejuni* CDT (Cj-CDT) and membrane cholesterol-rich microdomains and the role of cholesterol in the Cj-CDT intoxication of host cells have recently been reported (Lin et al., 2011). However, the molecular basis of Cj-CDT association with the cell membrane and delivery of toxin subunits into cells remains uncertain. This review provides an overview of recent studies and advancements that address Cj-CDT-cell interactions and intracellular trafficking pathways at the molecular level. Additionally, we discuss several novel strategies for developing CDT into a cancer therapeutic and its potential clinical applications.

## Composition and functions of Cj-CDT

The discovery of Cj-CDT and subsequent reporting of its biological function has continued for decades (Johnson and Lior, 1988; Mizuno et al., 1994). Evidence demonstrated that Cj-CDT comprises three protein subunits, Cj-CdtA, Cj-CdtB, and Cj-CdtC, encoded by an operon composed of the genes *cdtA, cdtB*, and *cdtC* (Pickett et al., 1996). Each *cdt* variant contains a consensus ribosome-binding site and encodes protein subunits with predicted molecular masses of 30,116, 28,989, and 21,157 kDa, respectively (Pickett et al., 1996). Among the three subunits, Cj-CdtA and Cj-CdtC are required for assembling a tripartite complex with Cj-CdtB for holotoxin activity (Lara-Tejero and Galan, 2001) and binding to the plasma membrane of cells (Bag et al., 1993). Cj-CdtB does not bind to the cell membrane, but rather possesses type I deoxyribonuclease (DNase I) activity, which causes DNA double-strand breakage (DSB) and leads to cell-cycle arrest at the G2/M phase, thereby inducing cell distention and resulting in senescence or target-cell death (Whitehouse et al., 1998; Lara-Tejero and Galan, 2000). In addition to its DNase I activity, the enzymatic subunit Aa-CdtB has phosphatidylinositol 3-4-5 trisphosphate (PIP3) phosphatase activity that induces apoptosis in T cells (Shenker et al., 2007). Structural and sequence analyses of Aa-CdtB indicated that it shared homology with inositol polyphosphate 5-phosphatase (Shenker et al., 2007). Moreover, mutation of Aa-CdtB reduces its phosphatase activity, abolishes CDT-induced G2/M arrest, and decreases DNase I-like activity. The phosphatase activity of CdtB may target Wee1 kinase or Cdc25 phosphatase that consequently alter cell cycle regulatory networks (Pickett and Whitehouse, 1999). These results are supported by a previous report which stated that the catalytic residue in Aa-CdtB possesses both DNase I and phosphatase activities (Dlakic, 2001). However, the phosphatase activity of Cj-CdtB has not yet been reported (Gargi et al., 2012).

The intoxication of lymphocytes by Aa-CDT results in PIP3 depletion and perturbation of phosphatidylinositol-3-kinase (PI-3K)/PIP3/Akt signaling concomitant with decreases in GSK3β phosphorylation (Shenker et al., 2016). Moreover, blockade of PI-3K signaling by Aa-CDT induces the production of pro-inflammatory cytokines such as IL-1β, TNFα, and IL-6 by macrophages (Shenker et al., 2014). These results indicate that CDT functions as an immune modulator. Similar results were found in a study showed that Cj-CDT induces IL-8 production and promotes chemotaxis by leukocytes, therefore induces inflammation in host intestines (Hickey et al., 2000). Moreover, *C. jejuni* with CDT enhances invasiveness in SCID mouse tissues at a level higher than that in *cdtB* mutants (Purdy et al., 2000). An NF-κB-deficient mouse infection model has confirmed the critical role of Cj-CDT in bacteria-inducing inflammatory responses and the contribution of Cj-CDT to persistent bacterial infection in hosts (Fox et al., 2004). Although its exact function in *C. jejuni*-induced pathogenesis has not been fully demonstrated, Cj-CDT definitely possesses critical roles in the invasiveness and modulation of immune response. Similar to Cj-CDT, a variety of pathogenic gram-negative bacteria have been identified as possessing CDT, including *Aggregatibacter actinomycetemcomitans* (Aa-CDT) (Ohara et al., 2004), *Escherichia coli* (Ec-CDT) (Johnson and Lior, 1988), *Haemophilus ducreyi* (Hd-CDT) (Cope et al., 1997), *Haemophilus parasuis* (Hp-CDT) (Zhang et al., 2012), *Helicobacter hepaticus* (Hh-CDT) (Young et al., 2000), and *Shigella dysenteriae* (Sd-CDT) (Okuda et al., 1995).

## Binding of Cj-CdtA/-CdtC to lipid rafts

CdtA and CdtC serve as carriers for delivering the active subunit, CdtB, into host cells (Nesic and Stebbins, 2005). CdtB is subsequently internalized, while CdtA and CdtC remain associated with the membrane receptor (Lee et al., 2003). The crystal structure of Hd-CDT revealed that CdtA and CdtC adopt lectin-type structures that are homologous to the plant toxin ricin (Nesic et al., 2004). The holotoxin contains two critical binding elements: an aromatic patch in CdtA and a deep groove at the interface of CdtA and CdtC (Nesic et al., 2004). Structure-based mutagenesis demonstrated that mutations of the aromatic patch and deep-groove residues inhibited toxin binding to the cell surface, followed by attenuation of cell intoxication, indicating that the two ricin-like lectin domains in CdtA and CdtC are critical for CdtB binding (Nesic and Stebbins, 2005).

**Figure 1:**
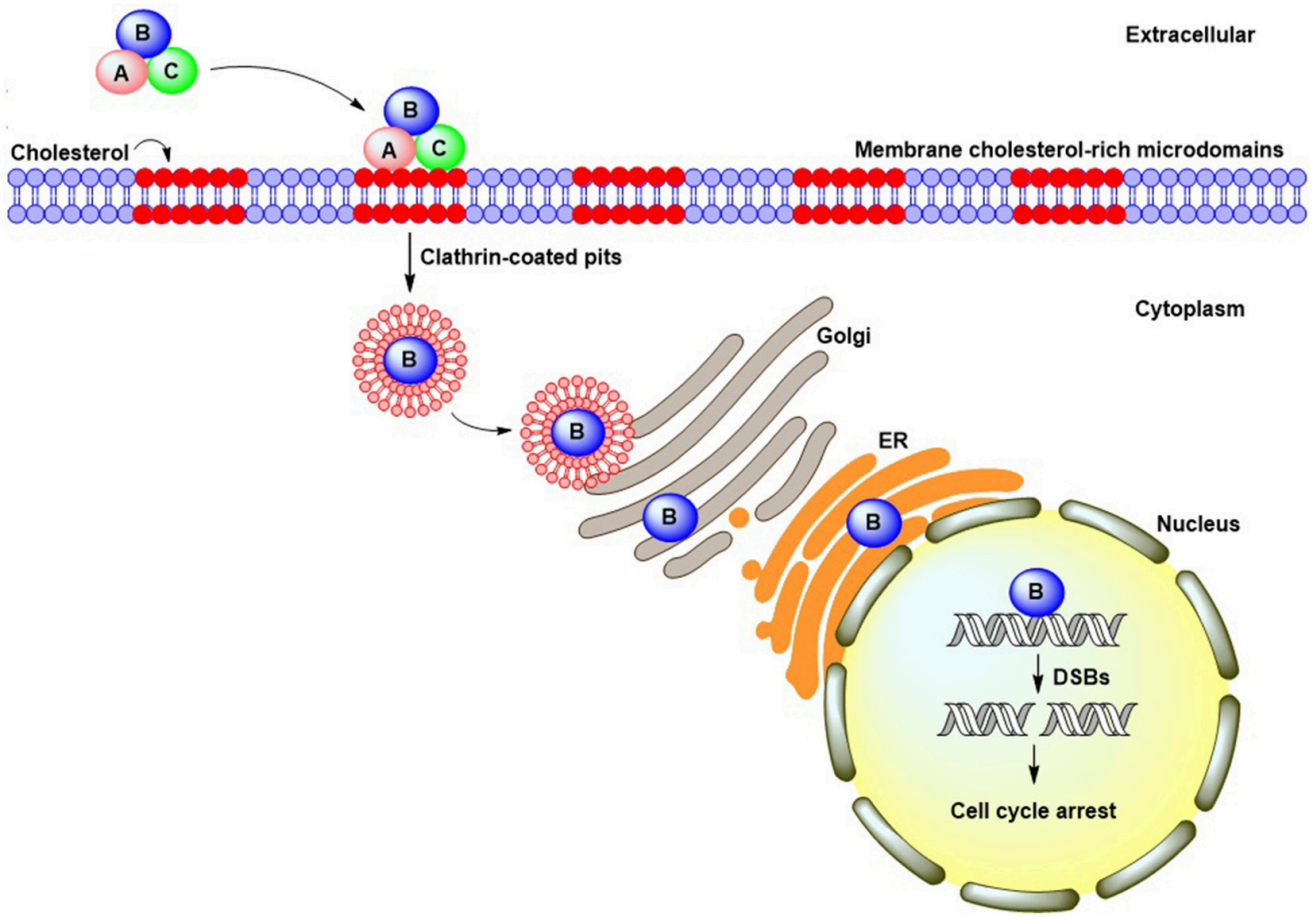
**CDT cellular intoxication pathway**. CdtA and CdtC bind to cell membrane cholesterol-rich microdomains and facilitate CdtB entry into cells through clathrin-coated pit endocytosis. Following internalization, CdtB translocates to the cytosol and may undergo retrograde trafficking from the Golgi complex into the endoplasmic reticulum. The CdtB subunit ultimately translocates to the nucleus by virtue of a putative nuclear localization signal present in its amino-acid sequence. Once in the nucleus, CdtB induces DNA double-strand breaks, which lead to cell-cycle arrest.

An analysis of Aa-CDT revealed that CdtA and CdtC were both bound to the cell membrane and associated with lipid rafts (Boesze-Battaglia et al., 2006), which contain abundant cholesterol, phospholipids, and sphingolipids (Brown and London, 1998). The structure of lipid rafts can be perturbed by the cholesterol-depletion agent methyl-β-cyclodextrin (MβCD) (Simons and Toomre, 2000). Treatment of cells with MβCD attenuated the binding activity of Hd-CDT (Guerra et al., 2005) and Aa-CDT (Boesze-Battaglia et al., 2006) to HeLa and Jurkat cells, respectively, and reduced cell intoxication. Our previous report demonstrated that Cj-CDT associated with cholesterol-rich microdomains in CHO-K1 cells in a modality similar to that observed in Hd-CDT and Aa-CDT (Lin et al., 2011). The co-localization of Aa-CDT and Cj-CDT with the lipid-raft markers ganglioside GM1 and caveolin-1, respectively, was observed by confocal microscopy (Boesze-Battaglia et al., 2006; Lin et al., 2011). More recently, we showed that Cj-CDT administration induced severe intestinal inflammation in mice fed a high-cholesterol diet relative to control mice fed a normal diet, demonstrating that cholesterol plays a crucial role in facilitating CDT-induced pathological derangement *in vivo* (Lai et al., 2015). These results collectively indicated that, similar to Hd-CDT and Aa-CDT, cholesterol provides an essential ligand for Cj-CDT binding to the cell membrane followed by host intoxication (Figure 1), and that CdtA and CdtC contain a cholesterol-binding motif.

## Cj-CdtC contains a conserved cholesterol recognition/interaction motif

A previous study reported the existence of a common cholesterol recognition/interaction amino-acid consensus (CRAC) pattern containing a conserved motif [L/V(X)_1–5_Y(X)_1–5_R/K] shown to interact with cholesterol (Li and Papadopoulos, 1998). It has been demonstrated that Aa-CdtC contains a CRAC region, which contributes to the association between toxin subunits and cholesterol (Boesze-Battaglia et al., 2009). The CRAC motif also exists in the CdtC subunit of *H. parasuis* (Zhou et al., 2012), and another study found CRAC-like region ^77^LPFGYVQFTNPK^88^ present in Cj-CdtC (Lin et al., 2011). A structure-based simulation indicated that the 12-amino acid residues of CRAC-like regions created a hydrophobic groove that contributes to Cj-CdtC hydrophobic interactions and hydrogen bonding with cholesterol (Lai et al., 2013). Mutation within the CRAC-like region (CdtC^Y81P^) reduced Cj-CDT binding to cells, but did not disrupt formation of the holotoxin complex (Lai et al., 2013). These reports were in agreement with studies of Aa-CdtC and Hp-CdtC (Boesze-Battaglia et al., 2009; Zhou et al., 2012), establishing that the CRAC-like region of Cj-CdtC plays a critical role in CDT-holotoxin binding to membrane cholesterol, but not in intermolecular interactions between each toxin subunit.

The results of a recent report showed that CdtA and CdtC derived from *E. coli* and *H. ducreyi* likely interact with distinct receptors on the cell surface and are independently sufficient to support CdtB-mediated cytotoxicity (Dixon et al., 2015). Depletion of cholesterol by MβCD reduces intoxication by Ec-CdtAB and Ec-CDT holotoxin, which suggests that Ec-CdtA can interact with cholesterol or a component(s) in lipid rafts to promote efficient intoxication (Dixon et al., 2015). This finding is similar to that of our previous study, which showed that membrane cholesterol is critical for Cj-CdtA binding and Cj-CDT intoxication in host cells (Lin et al., 2011). However, the receptors (i.e., cholesterol or components of cholesterol-rich microdomains) for Cj-CdtA require further confirmation.

Although the Cj-CdtC CRAC motif is important to holotoxin binding to membrane cholesterol, the cell-binding activity of a Cj-CdtC variant containing a mutation in this region was not thoroughly impaired. This result indicated that Cj-CdtC binding to the cell membrane was mediated by either cholesterol or non-cholesterol receptors (Lai et al., 2013). In a study of Aa-CDT, depletion of cellular cholesterol by MβCD did not alter CDT-binding in CHO-K1 cells (Damek-Poprawa et al., 2012). Interestingly, Cj-CDT intoxication of CHO-K1 cells was unchanged following cholesterol loading; however, it was enhanced following inhibition of *N*-linked glycosylation (Eshraghi et al., 2010). It was reported that Ec-CDT interaction with membrane glycoproteins in HeLa cells involved *N*-linked, but not *O*-linked fucose (McSweeney and Dreyfus, 2005). In addition, ganglioside GM3 served as a receptor for Aa-CDT (Mise et al., 2005). Interestingly, inactivation of sphingomyelin synthase 1 (*SGMS1*), which encodes an enzyme for sphingomyelin biosynthesis, abolished CDT intoxication by Aa-CDT, Cj-CDT, Ec-CDT, and Hd-CDT (Carette et al., 2011). Although these reports provided some conflicting results, Cj-CDT association with cholesterol and carbohydrates appears crucial for toxin-related triggering of the molecular machinery involving lipid rafts and enhancing the efficiency of toxin-related activity in target cells.

## Delivery of Cj-CDT into cells

Several gram-negative bacteria employ outer-membrane vesicles (OMVs) for delivering active proteins or toxins into host cells (Kulp and Kuehn, 2010). CDT associated with OMVs secreted from *C. jejuni* was first reported by Lindmark et al. (2009). Immunogold-labeling electron microscopy revealed that all three CDT subunits were tightly associated with OMVs, which functioned as a route for *C. jejuni* delivery of toxins into the cellular environment (Lindmark et al., 2009). Interactions of OMVs with membrane rafts have been reported in some bacterial pathogens (Kesty et al., 2004; Kaparakis et al., 2010). For example, CDT-associated OMVs secreted by *A. actinomycetemcomitans* can be delivered to the cytoplasm by membrane fusion dependent upon lipid rafts (Rompikuntal et al., 2012). Using confocal microscopy to track CdtB in cells treated with *A. actinomycetemcomitans* OMVs, nuclear translocation of the toxin-activating subunit was observed (Rompikuntal et al., 2012). The internalized *A. actinomycetemcomitans* OMVs then induced NOD1- and NOD2-dependent NF-κB activation (Thay et al., 2014). Similarly, a study of *C. jejuni* revealed that treatment of cells with MβCD partially inhibited OMV-induced inflammation, indicating that membrane rafts provided a certain role for *C. jejuni* OMV-mediated signal transduction (Elmi et al., 2012). These findings suggest that OMV interactions with lipid rafts may promote the delivery of Cj-CDT to target cells and enhance the efficiency of cell intoxication. Further investigations are needed to fully comprehend the extent to which Cj-CDT-associated OMVs rely upon membrane rafts for cellular internalization into the cytoplasm and nuclear translocation.

## Does Cj-CdtB bind to cholesterol?

Several reports indicated that CdtB alone binds to neither the cell membrane nor lipid rafts (Lara-Tejero and Galan, 2000; Guerra et al., 2005; Boesze-Battaglia et al., 2009). However, a recent study revealed that Aa-CdtB is capable of binding to large unilamellar vesicles containing cholesterol (Boesze-Battaglia et al., 2015). The authors demonstrated that, similar to Aa-CdtC, Aa-CdtB contains a CRAC motif that contributed to cholesterol binding and was required for toxin internalization and subsequent intoxication of both lymphocytes and macrophages (Boesze-Battaglia et al., 2015). However, in our Cj-CdtB study, this subunit was not observed interacting with membrane cholesterol in the absence of associations with CdtA and/or CdtC (Lin et al., 2011). Despite variations in the CdtB amino-acid sequence and structural characteristics among bacterial pathogens, the residues associated with DNase I and phosphatase activities are highly conserved (Shenker et al., 2007; Jinadasa et al., 2011; Gargi et al., 2012).

## Cj-CdtB nuclear translocation

Upon binding to lipid-membrane microdomains, CDT is rapidly internalized by clathrin-dependent endocytosis, which was found in *H. ducreyi* (Cortes-Bratti et al., 2000). Subsequent nuclear translocation requires Hd-CDT transport by a retrograde pathway from the trans-Golgi network (TGN) to the endoplasmic reticulum (ER) (Guerra et al., 2005). This modality is similar to other bacterial toxins secreted from different pathogens, including the Shiga toxin (Sandvig et al., 2010) and cholera toxin (Wernick et al., 2010), which are transported through the TGN to the ER prior to entering the cytosol. However, unlike other toxins, Hd-CDT does not exploit the mechanism of ER-associated degradation (ERAD) normally employed for translocation of misfolded proteins from the ER to the cytosol for proteasomal degradation (Guerra et al., 2009). Additionally, Hd-CdtB does not appear in the cytosol of intoxicated cells, suggesting that Hd-CdtB may translocate directly from the ER to the nucleus (Guerra et al., 2009). Interestingly, a recent study demonstrated that three important components of ERAD machinery, Derlin-2 (Derl2), E3 ubiquitin-protein ligase (Hrd1), and the AAA ATPase p97, are required for cell intoxication by CDT (Eshraghi et al., 2014). The authors also revealed that Derl2 deletion resulted in resistance to CDT-induced cell death by Aa-CDT and Hd-CDT, suggesting that the ERAD pathway is important for CDT retrotranslocation. However, cells lacking Derl2 displayed only modest resistance to Cj-CDT. Similar to Der2-deficient cells, cells deficient in Hrd1 were sensitive to Cj-CDT intoxication (Eshraghi et al., 2014). These findings suggest that Cj-CDT evolves distinct strategies to reach the host nucleus and harbors peculiar host requirements that differ from those of other CDTs.

CdtB possesses a putative nuclear-localization signal (NLS) that is critical for directing the toxin-activating subunit from the ER to the nucleus. By employing a domain-swapping experiment with an SV40T NLS, which has demonstrated that the Aa-CdtB subunit comprised two domains: an N-terminal domain for nuclear transport and a C-terminal activation domain (Nishikubo et al., 2003). McSweeney and Dreyfus demonstrated that two potential NLS sequences are present in the C-terminal region of Ec-CdtB-II (McSweeney and Dreyfus, 2004). In cells transfected with an Ec-CdtB-II-NLS1 mutant and Ec-CdtB-II-NLS1/2 double mutants, the intoxication effects of the subunits were abolished. These findings revealed that the putative NLS sequence(s) are important for CdtB intracellular trafficking and translocation to the nucleus. However, the presence of the NLS sequence(s) in Cj-CdtB has yet to be determined.

Although CDTs from pathogens other than *C. jejuni* are capable of retrograde transport through the TGN to the ER and ultimately into the nucleus, two critical issues have emerged regarding intracellular Cj-CDT trafficking. First, Cj-CDT neither relies on Derl2 nor requires Hrd1 for this activity (Eshraghi et al., 2014). Second, there is no experimental evidence regarding the role of NLS in Cj-CdtB. Future studies exploring specific components of Cj-CDT that exploit the ERAD pathway may help delineate the mechanism of Cj-CdtB nuclear translocation.

## Application of CDT in cancer therapy

Multidrug resistance is among the most critical reasons for treatment failure in cancer patients. One example of therapeutic resistance involves the presence of a major molecular efflux pump in cell membranes that enables cancer cells to bypass drug toxicity or alter cellular processes between the cytoplasm and the nucleus (Szakacs et al., 2006). It is reasonable to develop effective bacterial toxins as potential alternatives to treating refractory tumors, given that these toxins are easily obtained from bacteria and the bacteria can efficiently gain access to target cells through membrane-receptor internalization. Several bacterial virulence factors have been applied in clinical settings for cancer therapy, including the anthrax toxin from *Bacillus anthracis* (Liu et al., 2000), diphtheria toxin from *Corynebacterium diphtheriae* (Frankel et al., 2002), and Shiga toxin from *S. dysenteriae* and *E. coli* (Ishitoya et al., 2004). These studies provide evidence of the value in developing bacterial toxins as potential cancer therapeutic agents.

CDT is capable of inducing cell-cycle arrest by activating ataxia telangiectasia mutated (ATM)-dependent DNA-damage checkpoint responses and DSBs, similar to pathways induced by ionizing radiation (IR; Fahrer et al., 2014). Given this CDT function as a radiomimetic agent, emerging and effective therapeutic modalities have been tested as treatments for several cancer types (Table 1). This idea was demonstrated in our recent study where use of a combination of Cj-CDT and IR dramatically increased the efficacy of radiotherapy in radio-resistant prostate cancer cells (Lai et al., 2014a). Our results also revealed that Cj-CDT enhanced radio-sensitization attributable to the attenuation of DSB repair, long-term cell-cycle arrest in G2/M phase, and activation of the apoptotic pathway, indicating that Cj-CDT may be a potent therapeutic agent for radio-resistant cancer. Another study described local delivery of an Aa-*cdtB*-expressing plasmid into human gingival squamous carcinoma by sonoporation inhibiting the growth of tumor cells (Iwanaga et al., 2007). Moreover, a mutant Aa-CdtA variant conjugated to an anti-human CD133-monoclonal antibody led the toxin targeting to CD133^+^ head and neck squamous-cell carcinomas (Damek-Poprawa et al., 2011). This study provided evidence that genetically modified CDT could specifically target cancer stem cells and inhibit growth. Another study utilized Hd-CdtB fused with *B. anthracis*-toxin lethal factor to efficiently deliver toxin to various human tumor-cell lines, resulting in impressive anti-tumor effects in a murine experimental model (Bachran et al., 2014). Additionally, our recent report showed that replacing Cj-CdtA/-CdtC subunits with chitosan/heparin nanoparticles and encapsulating CdtB achieved markedly increased inhibition of gastric-cancer growth *in vitro* and *in vivo* without intoxication of primary gastric-epithelial cells (Lai et al., 2014b). This strategy (combination nanoparticle therapy) resulted in significant anti-tumor activities. The advantages of these strategies include avoidance of toxins poisoning normal cells by preferentially killing cancer cells. Therefore, CDT combined with multiple delivery approaches constitutes a potential anticancer drug that should be considered for further development.

**Table 1:** **Use of bacterial CDT variants for the experimental treatment of cancer**.

**Bacterium harboring CDT**	**Cancer cell type**	**References**
*A. actinomycetemcomitans*	Human gingival squamous carcinomas	Iwanaga et al., 2007
	Human head and neck squamous cell carcinomas	Damek-Poprawa et al., 2011
*C. jejuni*	Human prostate cancer	Lai et al., 2014a
	Human gastric cancer	Lai et al., 2014b
*H. ducreyi*	Human cervical carcinoma	Bachran et al., 2014
	Human head and neck squamous cell carcinomas	Bachran et al., 2014
	Human colon carcinoma	Bachran et al., 2014
	Human lung carcinoma	Bachran et al., 2014

## Utilization of CDT in clinical settings

FcεRI crosslinked with IgE activates PI-3K and generates PIP3, which plays a critical role in the activation of mast cells and degranulation, leading to allergic responses, and inflammation (Tkaczyk and Gilfillan, 2001). Aa-CdtB possesses PIP3-like activity and functions as an immunotoxin (Shenker et al., 2007). By fusing Aa-CdtB with the IgE Fcε-binding region, PI-3K/PIP3 signaling is disturbed, thereby inhibiting mast-cell degranulation (Shenker et al., 2010). These studies implicated CdtB as a promising targeted therapeutic agent for mast-cell-mediated diseases (Shenker et al., 2011). Unlike CdtB, the major functions of the CdtA/CdtC subunits involve binding to cholesterol-rich microdomains in the plasma membrane. Formation of a Hd-CdtA-CdtC noncovalent complex protected cells against CDT-holotoxin-indued cell death (Deng and Hansen, 2003). One potential explanation is that the Hd-CdtA-CdtC complex may occupy the holotoxin-biding region, which prevents or delays CDT-related cell death. Although the precise mechanism remains unclear, this study revealed a possible application of CDT subunits in the prevention of pathogenic infections requiring membrane-associated lipid rafts.

## Conclusions and future perspectives

Recent studies have demonstrated that Cj-CdtA/-CdtC interacts with membrane-associated lipid rafts, enabling the Cj-CdtB subunit to cross the cell membrane, undergo intracellular trafficking, and translocate to the nucleus. Several relevant issues have emerged and require further exploration, including (1) which molecule(s) in the lipid rafts serve as receptor(s) for Cj-CdtA/CdtC binding; (2) is the CRAC motif present in the Cj-CdtA subunit and what is its precise role in cholesterol binding; (3) to what extent do Cj-CDT-associated OMVs rely on membrane rafts for cellular internalization and nuclear translocation; (4) does the Cj-CdtB subunit contain an NLS sequence; and (5) does controlling cholesterol prevent CDT intoxication *in vivo*. Despite the availability of genetic information and experimental studies, the understanding of Cj-CDT-associated activities at the molecular level remains incomplete.

Current experimental and epidemiological studies support critical roles of CDT in *C. jejuni*-induced pathogenesis, including cell adhesion, invasion, and inflammation. Bacteria that cause persistent infections associated with chronic inflammatory responses may possess a high risk of promoting carcinogenesis (Grivennikov et al., 2010). However, neither *C. jejuni* nor its CDT proteins are associated with malignancies in the gastrointestinal tract (Brauner et al., 2010). Although rare studies reported CDT capable of inducing tumorigenesis, CDT-producing *Helicobacter bilis-* and *H. hepaticus*-infected mice developed colon and hepatic cancers, respectively (Ericsson et al., 2010; Fox et al., 2011). These reports revealed a potential correlation between CDT and carcinogenesis (Guerra et al., 2011). Considering the biological safety and suitability of developing Cj-CDT as an anticancer therapeutic agent, *in vivo* investigation is necessary to determine the existence of a correlation between chronic infection by CDT-producing *C. jejuni* and increases risk of cancer development. Future investigations focusing on both *in vitro* and *in vivo* models will likely unveil CDT-host interactions at the molecular level and develop novel strategies to control *C. jejuni* infection. The innovative technologies developed because of this research will enable utilization of this genotoxin as a cancer therapeutic agent.

## Author contributions

Conception or design of this work: HL, J-TH, C-HL. Drafting the manuscript: C-KL, Y-AC, C-JL, H-JL, M-CK. Revising this article critically for important intellectual content: M-ZH, Y-HL, CC-N, C-JC. Final approval: U-GL, L-CL, C-HL.

### Conflict of interest statement

The authors declare that the research was conducted in the absence of any commercial or financial relationships that could be construed as a potential conflict of interest.
